# Empowering voice assistants with TinyML for user-centric innovations and real-world applications

**DOI:** 10.1038/s41598-025-96588-1

**Published:** 2025-05-02

**Authors:** Sireesha Chittepu, Sheshikala Martha, Debajyoty Banik

**Affiliations:** 1 School of CS & AI, SR University, Warangal, India; 2 School of Engineering, Anurag University, Hyderabad, India

**Keywords:** TinyML, Voice assistant, Deep learning, Trending technologies, Aerospace engineering, Biomedical engineering, Nanoscience and technology

## Abstract

This study explores the motivations behind integrating TinyML-based voice assistants into daily life, focusing on enhancing their user interface (UI) and functionality to improve user experience. This research discusses real-world applications like smart home automation, visually impaired assistive technologies, and healthcare monitoring. This review acknowledges various problems and helps us understand why TinyML exerts such significant implications in numerous domains. Researchers derive solutions from this study on how voice assistants integrated with TinyML can effectively analyze and adjust to user behaviour patterns in real-world scenarios, thereby enabling the delivery of dynamic and responsive content to enhance user engagement. The article also focused on limitations while implementing TinyML. Researchers will understand the detailed issues that are unavailable in most papers. This work explores features that can be embedded in voice assistants, like smart home automation, smart watches, smart glasses for visually impaired people, etc., using TinyML. A comparative review of current methods identifies areas of research gaps such as deployment difficulties, noise interference, and model efficiency on low-resource devices. From this study, researchers can directly identify the research gap with minimal effort, which may motivate them to focus more on solving the open problems due to optimize the problem identification time.

## Introduction

TinyML-based voice assistants enhance everyday life by improving user interfaces and real-world functionalities, While researchers focus on improving user adoption of voice assistants, fewer studies explore their integration with emerging technologies for expanded functionalities, such as smart home automation and healthcare monitoring. This voice assistant system helps consumers in various ways^1^; in smart home assistants^2^; in Smart Healthcare^3^, etc. Researchers were also able to identify depression detection using multi-modal techniques using Speech and EEG signals^4^. Artificial Intelligence (AI) is being applied in a wide range of research fields and has been shown to be an innovative tool for solving a wide range of research issues. Nevertheless, there is a cost^5^ associated with the massive processing needed to train AI systems. Driven by the need to lower the cost, carbon footprint, and energy use of the machines running ML techniques, TinyML is now seen as a viable alternative to artificial intelligence that focuses on applications and technology for incredibly low-profile devices. There are several exciting fields in which TinyML can have a significant influence. Anomaly detection plays a crucial role in industry by helping to minimize delays for repairs and boosting production efficiency. By implementing machine learning algorithms at the edge, it is feasible to continually monitor and evaluate the noise that the machine produces while it is in operation, which may indicate a potential malfunction. Real-time analysis of various parameters, such as noises or vibrations, can assist in saving time while replacing or fixing faulty equipment without causing further delays. The Web of Animals is one of the most recent study areas in the environment whereby sensors have been widely used. Most researchers still need help understanding animal behaviour. Studying animal behavior through continuous observation for short periods can be a challenging practice. The IoT, particularly TinyML, can significantly eliminate the need for this laborious work. Gaining more in-depth understanding of animal life and anticipating potential dangers might be beneficial. An elephant is fitted with a collar in the elephant’s TinyML project uses GPS to track the elephant’s movements in real-time. The implanted sensors gather pictures of its surroundings, which TinyML continually processes and analyzes to forecast occurrences surrounding each animal. While a motion sensor is utilized to assess the elephant’s movement further, other machine-learning models may also be employed to comprehend and detect the elephant’s mood. TinyML opens up new options and provides fresh perspectives on sustainable development. TinyML lowers latency so real-time applications, such as voice and picture recognition, may be implemented at the data source. Additionally, TinyML models may function without an internet connection-something that is not possible in a cloud environment. Because TinyML processes data without requiring it to leave the device, it dramatically enhances user privacy and conforms with data protection laws. The scope of the paper is to present more information about the TinyML feature with voice assistant, which is embedded in small devices with IoT.

Although TinyML provides considerable benefits, including low power usage, low latency, and increased privacy, it also comes with some trade-offs compared to cloud-based systems. One of the biggest drawbacks is the lower computational capability of microcontrollers, which limits the complexity of models that can be used. In contrast to cloud-based voice assistants that utilize large computational resources for deep learning, TinyML-based systems have to depend on optimized, light models that strike a balance between accuracy and efficiency. TinyML devices also come with limited memory and storage, which creates difficulties in dealing with large datasets and real-time adjustment. Yet these trade-offs are offset by advantages like enhanced data security-because processing is local instead of on remote servers-and reduced energy use, which makes TinyML ideal for edge applications in resource-scarce environments.

### Research aim and hypothesis

This study aims to evaluate the effectiveness of TinyML-based voice assistants by analyzing key performance metrics, including accuracy, computational efficiency, and power consumption, across different hardware platforms and deployment scenarios. Hypothesis TinyML-based voice assistants can achieve comparable accuracy to cloud-based systems while significantly reducing energy consumption and enhancing user privacy, making them viable alternatives for real-world applications.

## Research questions

The primary research questions which are framed to understand the TinyML-based voice assistants concept more detailed manner is shown bellow:

1. What are the main implementation problems for TinyML-based voice assistants in comparison with conventional cloud-based systems?
2. How can TinyML-based voice assistant performance and deployment affect various hardware platforms under varying noise levels?
3. How do ambient conditions and background noise impact the performance and reliability of TinyML-based voice assistants?
4. Based on dataset comparisons, which TinyML model architecture balances accuracy, computational efficiency, and real-world feasibility?

## Voice assistant in TinyML

Traditional voice assistants rely on cloud-based processing, which results in latency, high energy consumption, and privacy concerns. TinyML enables on-device inference, reducing cloud dependency. However, limitations include reduced computational power and memory constraints. TinyML offers a promising alternative by enabling on-device inference, reducing the need for cloud connectivity. However, this transition also introduces challenges, such as lower model complexity and constrained memory resources.

Table 1 provides a comparative analysis of TinyML-based voice assistants and traditional cloud-based implementations across key performance metrics, including power consumption, latency, accuracy, privacy, and scalability.

**Table 1:** Comparison of TinyML-based and traditional ML-based voice assistants.

Metric	TinyML-based voice assistants	Traditional cloud-based voice assistants
Power consumption	Extremely low (mW range, runs on MCUs)	High (requires GPUs/CPUs, watts to kW)
Latency	Low (on-device processing, no cloud dependency)	High (network delays + cloud inference time)
Accuracy	Moderate (optimized models may slightly reduce accuracy)	High (large models leverage vast data resources)
Privacy & Security	High (local processing, no cloud data transfer)	Lower (data transmitted to cloud for processing)
Scalability	Ideal for embedded & IoT applications	Requires powerful hardware & internet access

The comparison highlights that while TinyML significantly improves energy efficiency and privacy, it faces limitations in computational power and accuracy compared to cloud-based solutions. Overcoming these trade-offs requires advances in model compression, noise robustness, and hardware optimization for embedded systems.

**Fig. 1:**
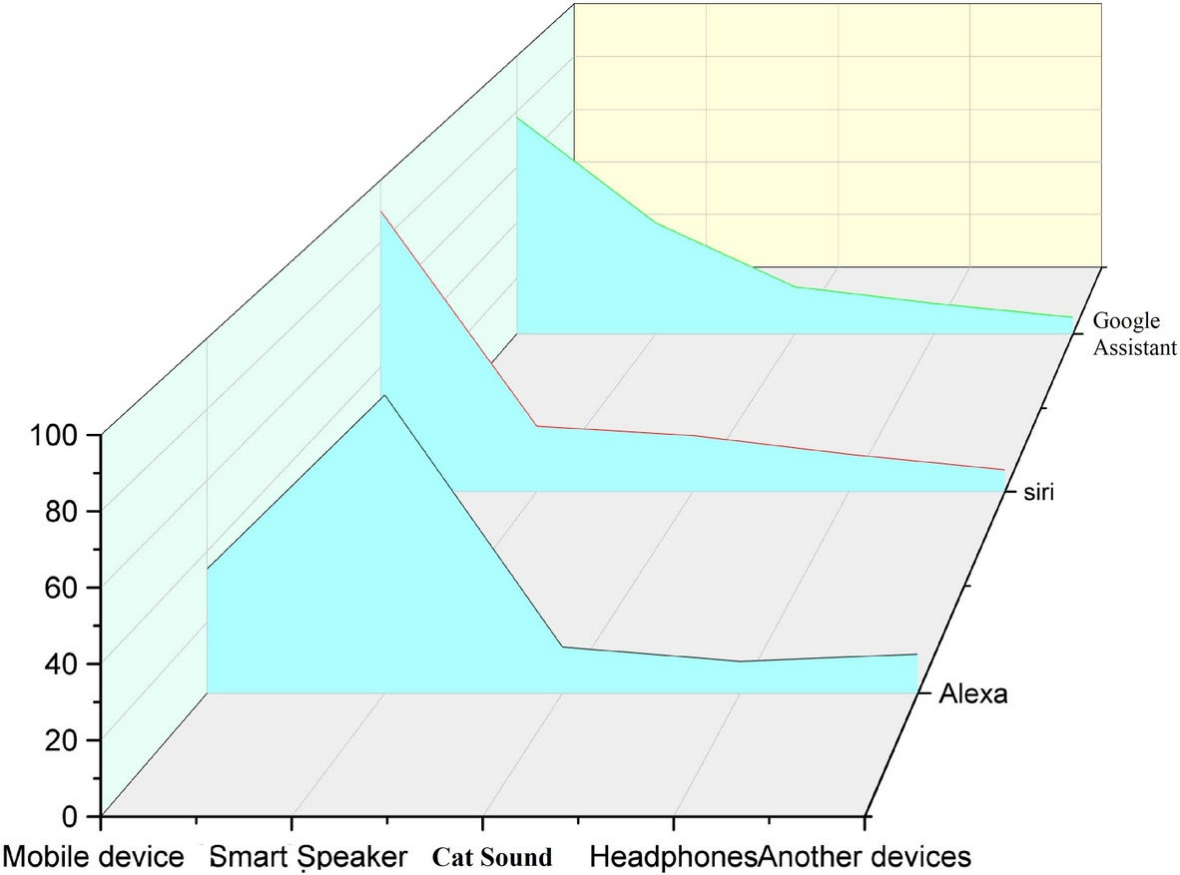
Distribution of voice assistant usage across different devices. $like Alexa, Siri, Google Assistant$ used in different devices like mobile devices, smart speaker, in the cars, in the headphones etc.

Voice assistants(VA) are the voice-enabled artificial intelligence^23^. Amazon’s Alexa, Apple’s Siri, Google Assistant, Microsoft’s Cortana, etc., are getting interest in the VA system. Figure 1 describes the statistics about which voice assistants are used in different devices like mobiles, smart speakers, cars, headphones, etc.; we can find that Siri is mostly used in mobiles, in smart speakers, Alexa is utilized, and in the cars, google assistants are frequently used. We can also notice that Siri is used in headphones frequently. A few voice assistants were taken, and they were tested and then calculated their mean(m) and standard deviation(s.d) were by considering the measurement values of functional intelligence, creativity, and protective quality. For all the voice assistants, various features like Information Quality, Correctness, Quality, Time per task, and Emotional Intelligence are mentioned in Table 2. Now, after all this analysis, we can understand that voice assistants in small wearable devices like smartwatches, fitness trackers, smart glasses, etc. Will be really very helpful on a regular basis if we embed these voice assistants in these devices using TinyML.

**Table 2:** Information of Google, Cortana, and Alexa statistics.

Voice assistant	Features	Citations
Google assistant	Information quality - 90.3%	^14^
	Quality - Excellent; Correctness - Excellent	^25^
	Time per task - 79.0 (49.0–116.0)	^13^
	Emotional intelligence (mean) - 3.90	^23^
Cortana	Trust - 93.8%	^14^
	Quality - Above average; Correctness - Above average	^25^
	Emotional intelligence (mean) - 4.36	^23^
Alexa	Personal innovativeness - 92.6%	^14^
	Quality - Excellent; Correctness	^25^
	Time per task (s) - 63.0 (41.3–106.5)	^13^
	Emotional intelligence (mean) - 3.77	^23^

TinyML can be used in healthcare for blood pressure monitoring, neural speech enhancement for hearing aids, improving wearable and ambulatory devices, tiny reservoir networks for the detection of pathological conditions, and We can also use TinyML-based voice assistants in smart farming. It can be used in crop management, smart irrigation, smart greenhouse, etc.; we can give some commands like stop, left, right, and train the machine learning model, and then we can easily make smart farming using 4.0 technologies. We can also include voice assistants based TinyML in vehicles to get information like path detection, parking assistance, object detection(and also classification, response), path change assistance etc., from the voice assistance we have deployed in vehicles. Information about the TinyML model, its accuracy, and various software tools are mentioned in Table 3. The cost analysis in Table 3 points out that TinyML voice assistants are much cheaper than cloud-based solutions and are well-suited for mass deployment in resource-limited settings. Nevertheless, practical deployment has a number of challenges. For instance, TinyML deployment on microcontroller units (MCUs) like the Arduino Nano 33 BLE Sense or ESP32 comes at the expense of processing power and model complexity. These devices have limited storage and RAM, and hence highly optimized models are needed to run effectively. Also, mass deployments in industrial automation or smart city scenarios need strong power management techniques to allow for uninterrupted running, since battery life is a severe limitation. Also, edge-based deployments might need firmware updates at regular intervals, which is difficult in distributed or remote environments. Solving these problems is key to effectively scaling TinyML-based voice assistants whileAmong all DNN model achieved 99% accuracy. Apart from this, a lot of filtering in the voices is required. For example, the same words are pronounced differently by different persons. This is because every person has distinct pitch and loudness; in^12^, Fourier transformation has been used to analyze the sound waves to get information regarding the frequency domain. In^12^, the author mentioned that FFT is a way to apply Fourier transformation. The Fast Fourier transform (FFT) is a computational procedure utilized to efficiently calculate the discrete Fourier transform of a given input, offering a notable speed improvement compared to direct computation. Equation (1) and (2) is the formulae for FFT. Equation (3) is FFT matrix, and Eq. 4) is FFT inverse matrix formel.

**Table 3:** Accuracy of different TinyML models and their software tools.

Model		Software tool	Accuracy
ProtoNN	EdgeML	93.58%	
CNN+GRU	CMIS-NN	85.4%	
TCN	NEMO/DORY	93.8%	
TCN	TFLite+GAPFlow	94.0%	
TCN	TFlite	94.0%	
TCN	CUBEAI+TFLite	94.0%	
RF	NA	94.5%	
Bonsal	EdgeML	94.2%	
DNN	Gestures dataset	TFLite	99%
DNN	Mnist dataset	TFLite	99%
SVM, logistic regression, decision tree, random forest	Gestures dataset	TFLite	95%
SVM, logistic regression, decision tree, random forest	Mnist dataset	TFLite	90.3%
CNN-LSTM-DNN	NA	93.5%

1 $$\begin{aligned} y_{k}&=\sum _{j=0}^{n-1} a_{j} \omega _{n}^{k j} \end{aligned}$$

2 $$\begin{aligned} \omega _{n}^{k j}&=e^{2 \Pi i / n} \end{aligned}$$

3 $$\begin{aligned} \begin{pmatrix} y_{o} \\ y_{1}\\ y_{2}\\ .\\ .\\ .\\ y_{n-1} \end{pmatrix}&= \begin{pmatrix} 1 & 1 & .& 1\\ 1 & \omega _{n}& .& \omega _{n}^{(n-1)}\\ 1 & \omega _{n}^{2}& .& \omega _{n}^{2(n-1)}\\ .& .& .& .& \\ .& .& .& .& \\ .& .& .& .& \\ 1 & \omega _{n}^{n-1}& .& \omega _{n}^{(n-1)(n-1)} \end{pmatrix} \begin{pmatrix} a_{o} \\ a_{1}\\ a_{2}\\ .\\ .\\ .\\ a_{n-1} \end{pmatrix} \end{aligned}$$

The inverse matrix, for instance, is extracted by replacing every $\omega _{n}^{k j}$ with $^{k j} / n$. In both cases, $n$ or $n_{F F T}$ and it is called FFT length.

4 $$\begin{aligned} a_{k}= & \frac{1}{n} \sum _{j=0}^{n-1} y_{j} \omega _{n}^{-k j} \nonumber \\ \omega _{n}^{-k j}= & e^{-2 \Pi i / n} \nonumber \\ \begin{pmatrix} a_{o} \\ a_{1}\\ a_{2}\\ .\\ .\\ .\\ a_{n-1} \end{pmatrix}= & \begin{pmatrix} 1 & 1 & .& 1\\ 1 & \omega _{n}& .& \omega _{n}^{(n-1)}\\ 1 & \omega _{n}^{2}& .& \omega _{n}^{2(n-1)}\\ .& .& .& .& \\ .& .& .& .& \\ .& .& .& .& \\ 1 & \omega _{n}^{n-1}& .& \omega _{n}^{(n-1)(n-1)} \end{pmatrix} \begin{pmatrix} y_{o} \\ y_{1}\\ y_{2}\\ .\\ .\\ .\\ y_{n-1} \end{pmatrix} \end{aligned}$$

While TinyML-based voice assistants face hardware constraints such as limited computational power, memory, and storage, emerging solutions are addressing these challenges. Newer microcontroller architectures, such as ARM Cortex-M55 with Ethos-U55 NPU and RISC-V-based MCUs, are designed to provide enhanced machine learning capabilities with improved efficiency. These architectures offer better power management and increased processing capacity, making them more suitable for deploying TinyML models at scale.

Due to their high computational complexity and memory requirements, transformer-based models such as BERT and GPT are still impractical for use in TinyML applications, even though their state-of-the-art performance in NLP and speech processing tasks. Transformers are inefficient for microcontroller units (MCUs) with limited resources because they rely on self-attention mechanisms that require large-scale parameter tuning and extensive matrix multiplications. The power and latency requirements needed for real-time inference on edge devices are difficult for even optimized versions like TinyBERT to meet. Conversely, TinyML applications give preference to lightweight models like CNNs, RNNs, and decision trees because they offer an adequate balance between computational viability, accuracy, and energy efficiency. In order to modify Transformer-based architectures for low-power TinyML platforms, future studies could investigate cutting-edge model compression strategies like quantization-aware training and knowledge distillation.

Furthermore, frameworks like TensorFlow Lite Micro (TFLM) play a crucial role in optimizing TinyML models for deployment on resource-constrained devices. TFLM enables quantization-aware training, reducing model size while preserving accuracy. It also supports hardware acceleration on compatible MCUs, significantly improving inference speed and energy efficiency. Other frameworks such as Edge Impulse and TinyMLgen further streamline the process by offering automated model conversion and deployment tools.

## RQ1: What are the main implementation problems for TinyML-based voice assistants in comparison with conventional cloud-based systems?

Nowadays, we are using a lot of AI algorithms, and simultaneously, the price of corresponding hardware to run those AI is increasing. The alternative of the cloud does not always overcome the cost issue because the computation time of the cloud is directly proportional to the processes being executed. The environment is also impacted by this; authors of^24^ mentioned that to prevent the effects that are caused by AI algorithms on the ecosystem, TinyML has been established as a cloud alternative. In terms of privacy, the data in TinyML has not to be sent to any cloud storage which automatically leads to data security. The cost of TinyML is low as we are sending any data to cloud storage, which leads to low energy consumption. In^26^, the author states that we are using small devices regularly in which TinyML has to be given more priority compared to the cloud because local ML tasks might become expensive compared to TinyML and also says that the cloud stores billions of data so, it becomes difficult to search a specific data in it whereas in TinyML, generally, it stores few data so that we can successfully search and find the data easily, the paper also mentioned that we can deploy the machine learning model easily into the TinyML as compared to cloud. So, we can notice that TinyML is less expensive compared to the cloud, and it does not affect the ecosystem. We can search and retrieve the data from TinyML easily compared to the cloud. It is easy to deploy machine learning models into TinyML compared to the cloud. This is the reason we prefer TinyML over the cloud. By Table 4, we find TinyML, which is economically preferred compared with the cloud.

**Table 4:** Different platforms cost based on their architecture as of 2024.

Platform	Architecture	Price
Cloud	GPU	9000 USD
Mobile	CPU	750 USD
TinyML	MCU	3 USD

**Fig. 2:**
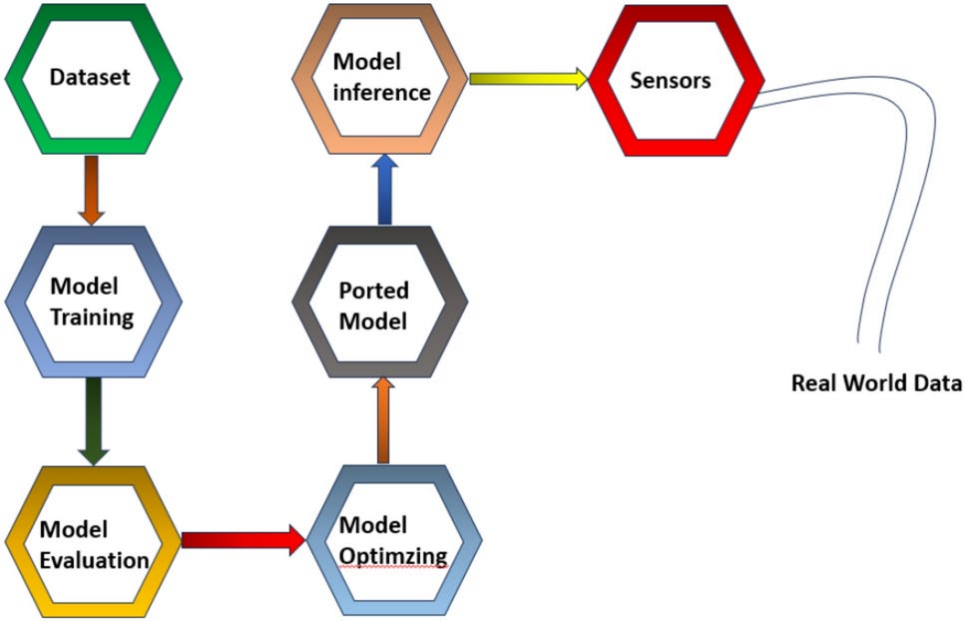
Step-by-step process of TinyML-based voice assistant deployment.

Sequential flow is shown in Fig. 2, is the sequence of the most important steps that one would follow while developing and deploying a solution to the problems in the domain of TinyML. Each step should be looked at while keeping in mind that it is necessitated by resource constraints-a predominant feature of TinyML applications in terms of memory, processing power, and energy availability.

## RQ2: How can TinyML-based voice assistance performance and deployment affect various hardware platforms?

Authors of^27^ focus on metrics and methods to assess TinyML, and here we come across the problem of deploying a machine learning model on TinyML. The paper also says that deploying traditionally causes traffic problems with massive data, which simultaneously results from the outperforming of support systems, so researchers have given a solution to filter data where we remove extra data from the storage. Hence, we ensure fewer traffic problems compared to before, and this process is data filtration. Researchers also noticed that the absence of a few frameworks makes the deployment process slow; we should also train the model along with the installation of frameworks. We can analyze the challenges faced by frameworks below.

- Installing models in many embedded systems is not easy and its portability is not so good.
- We ensure shortage in testing while performing real-world applications.
- Infrastructure to train every model to test it and then execute it is lacking.
- We have minimum features that support debugging.

Researchers suggest tensorflow Lite micro (TFLM) to overcome portability issues, and TFLM increases the flexibility in deploying the models so that we can easily embed them into the hardware. Authors of^28^ found the gap that while deploying, we train the model with a certain amount of data where we have some limited data. However, while using the model in real applications, the offline data we are giving may not match with the deployment environment. It is compared with a car example that when a car is driving, we need to adapt to the environment like landscape, slopes, etc.; we need to manage the car speed according to the surrounding environment; in the same way, the deployed model should adapt and match the environment. To overcome the challenges faced during deployment, as discussed above, the authors of^30^ gave a solution where we create a TinyCNN family for efficient deployment in microcontroller units, we introduce GPA as a module (SoM) system on the CNN interface, and then perform a closed-loop learning methodology, and then introduce the ML predictor to swap TinyCNN for runtime^30^, describes TinyML-based voice assistance performance and deployment effects on various hardware platforms. The closed loop learning method is where we first collect the data from the sensors, then train the model from the foundation level and then push the updates to the deployed model; from these methods, we can solve two research gaps: first is increasing the robustness of deployment and second is increasing the performance. Authors of^13^ have used DNAS to identify models which have an accuracy rate and which are contented to SRAM, flash, and other latency constraints. Now, we are going to briefly discuss DNAS and how it can be functional with ML model designs for microcontroller units. It consists of decision nodes; the output of this decision node varies from 1 to $K$ choices.

$$\begin{aligned} y=\sum _{k=1}^{K} z_{k} f_{k}\left( x, \theta _{k}\right) , \sum _{k=1}^{K} z_{k}=1 \end{aligned}$$

Here input tensor is (*x*), the function executed by value (*k*) is $(f_{k}())$ and the variables of the function are $(\theta _{k}, K)$ and these are total options for the decision model and $(z_{k} in {0,1})$ represents one of the $K$ values. Primary aim of this for selection of $$z = \left[ \begin{array}{lll}z\_{1}&\ldots&z\_{K}\end{array}\right]$$ to all decision nodes in the supernet. In the current work, we inhibit the width for each layer and the total depth. In such case every value $f_{k}()$ depicts a function with a distinct number of ways or identities.

According to^13^, DNAS might create a few models that do not match one or more microcontroller unit limits without any other model constraints. It is also given in^13^ that the final size of the model and activations done by modern neural networks, flash, and SRAM play crucial roles in model design. So, authors of^13^ concluded that we need to integrate a few standardized terms in DNAS methods to fit final models in flash and perform activations that can fit in SRAM. For model size considerations, we declare a specific selection size from supernet using :

$$\begin{aligned} \sum _{k=1}^{K} s_{k}\left| \theta _{k}\right| \end{aligned}$$

Here cardinality of $\theta _{k}$ is denoted by $\left| \theta _{k}\right|$. After summing each node size, we get the size of the supernet as an operation of decision parameters $z$ to every decision node for which we use regularization of the DNAS so that final models meet the micro controller unit’s eflash constraint.

To guarantee that final model satisfies SRAM constraints, authors of^13^ adopted working memory architecture, which declares that working memory required for node with the inputs $\left\{ x_{1}, \ldots , x_{N}\right\}$ and outputs $\left\{ y_{1}, \ldots , y_{M}\right\}$ is given by $\sum _{n-1}^{N}\left| x_{n}\right| +\sum _{m-1}^{M}\left| y_{m}\right|$. The entire working memory of each network node, in which it is included the DNAS operation so that the selected method or architecture meets the microcontroller’s SRAM constraints. Noise interference significantly affects the performance of TinyML-based voice assistants, making robust noise mitigation crucial. While several denoising techniques exist, their effectiveness varies across different conditions. Table 5 compare Automatic Speech Recognition (ASR)-based models, spectral subtraction, and deep learning-based denoising methods in terms of accuracy improvement and computational efficiency.

**Table 5:** Comparative analysis of noise mitigation techniques for TinyML-based voice assistants.

Technique	Accuracy improvement (%)	Computational cost	Latency	Power consumption	Hardware suitability	Deployment challenges
ASR-based models	10–12%	Medium	Low	Moderate	MCUs & DSPs	Requires well-trained noise suppression models
Spectral subtraction	6–9%	Low	Very Low	Very Low	Ideal for low-power MCUs	Less effective in non-stationary noise environments
Deep learning-based methods (SEGAN, CNNs)	15–18%	High	Medium-High	High	Requires advanced MCUs or Edge TPUs	High computational demand & energy usage

### Example

In a smartwatch, TinyML enables real-time voice command execution without requiring an internet connection, making it ideal for remote healthcare monitoring.

## RQ3: What effects do ambient circumstances and noise levels have on the functionality and reliability of TinyML-based voice assistants?

For the past few years, we are frequently using voice assistants like Alexa, Cortana, Siri, and Google Assistant, which generally give some commands and responses to us; every voice assistant performs three steps. first is speech recognition, i.e, we give some command like “Hi gooHow can TinyML-based voice assistance performance and deployment affect various hardware platforms? gle! what is the time now?” here the model takes keywords and recognizes the speech and second is natural language understanding, i.e., it understands the keyword and regarding information. Then third is giving a response to us according to the prompt. Authors of^31^ have discussed that voice assistants should grab users’ attention by giving some beep sound before giving the response, and voice assistants should maintain some privacy while giving responses, which should automatically detect whether the person is alone or not. Moreover, authors of^32^ described that Background noise significantly impacts voice assistant performance, leading to reduced speech recognition accuracy and misinterpretations. To address this issue, researchers have developed various noise mitigation techniques, each with trade-offs in computational efficiency and effectiveness. The researchers have given a denoising solution where sound enhancement algorithms have been introduced such that they recognize the voice in noisy conditions^32^. They have also used the ASR-based model, where researchers have tested the model with 36 noise backgrounds, and they have found that the accuracy of voice recognition is higher in the ASR-based model compared to the CNN model^33^ says that sound enhancement algorithms do not improve the outcomes of the clean voice data, but for noisy conditions, and a combination of SE and WUW^33^ improves the response.

**Table 6:** Different types of prototypes and algorithms used in that model which can used by visually impaired person.

Prototype	Functionalities	Algorithms
Helmet with omnidirectional camera and sensors(IMUs), backpack with laptop	Indoor positioning, tracking, and indoor scene recognition	Simultaneous localization and mapping algorithm (SLAM)
Stereo camera mounted on a helmet, smartphone, web application, and a cloud platform	Indoor positioning, object detection and recognition, OCR, Speech processing	Vision-based SLAM, Deep CNN based models
A head-mounted RGB-D camera, a laptop, a haptic feedback vest, a smartphone user interface, andIMU sensors	Indoor positioning and navigation with haptic feedback.	Vision based SLAM algorithm
Google Glass for capturing the image and server for object classification	Obstacles detection	AlexNet CNN model
Single camera mounted on Raspberry Pi board	Object detection and recognition, Distance estimation	YOLO object detection algorithm, Distance estimation algorithm

## RQ4: Which TinyML based voice assistant model has better approach based on dataset comparison?

**Fig. 3:**
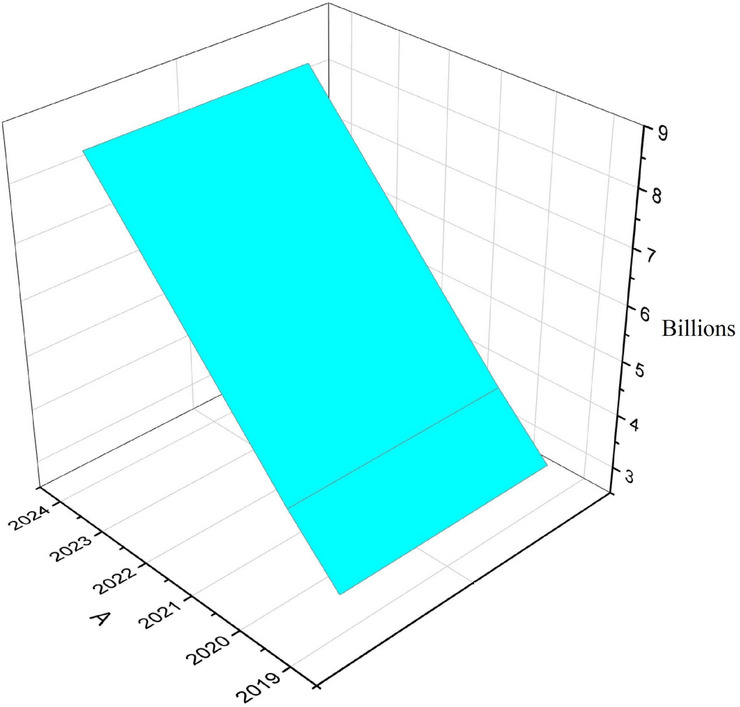
Worldwide statistics of voice assistant systems vs year.

For the comparison, a few datasets are collected in various domains (i.e., the tiny voice assistant in smart homes, watches, vehicles, for physically impaired persons). Firstly, we consider voice assistants in homes and how widely people are using them frequently 3 says that no.of users and purchases of voice assistants are increasing regularly because users tend to make their lives easy, by using these voice assistants like Alexa, google assistants etc., users are getting entertained because they listen songs from them just by giving prompt. They listen to the news by giving commands. In this way, voice assistants are making our lives more advanced and better. By this, we can understand the reason for increase the in uses worldwide since 2019 rapidly, which is noticed in Fig. 3.

Firstly we are comparing datasets of samples of human sounds, regular sounds, and disturbances in the home, and these samples are used in making effective home automation with the help of lively voice assistants. Using real-life examples, authors of^29^ have given information that voice assistants like Alexa perform better than any other AI assistants. Researchers also concluded that voice assistants will help in home automation; here, a few datasets of human intentions are collected, and they have observed that based on human intentions, the sounds that are coming from voice assistants can detect them, respond correspondingly like light issues, etc. They have put in a voice assistant that watches the home 24/7 through its cameras and understands the surroundings, and then it starts to control the home. The author of^29^ intends to create a lively voice assistant; it means that currently, we are using voice assistants that understand our voice commands such as “Hey Google! play music” then it plays music,. However, the author is aiming for creating lively assistant who understands the environment automatically and gives a response. Still, here many mismatches may arise this is a significant drawback however there are many advantages regarding this paper because many information regarding lively assistants are given such as human-computer interaction regarding this model and design engineering, design process are discussed very clearly. The author of^6^ also gave more information regarding Google Home and how we can deploy the lively model using TinyML. The author gave information about what hardware we need to use in this model, and it also gives information regarding the training of the model using different sound samples with tensorflow lite format^6^ has given more information regarding TinyML deployment and development of the model and its hardware, whereas this needs to be informed clearly in^29^ as it is done in^6^. So, a lively voice assistant model is perfect if it understands human emotions correctly because sometimes mismatches occur.

**Table 7:** Different voice assistants in smart watches and their input types.

Product name	Compatibility	Connectivity	Input types (incl. sensors)
Samsung Galaxy Watch 4	Google Assistant	Wi-Fi, Bluetooth, NFC, GPS	Microphone, barometer, accelerometer, gyroscope, optical heart rate sensor, electrical heart sensor, bioelectrical impedance analysis sensor, light sensor, geomagnetic sensor, hall sensor
Apple Watch S3	Apple Siri	Wi-Fi, Bluetooth, NFC, GPS	Microphone, force touch, barometric altimeter, optical heart rate, accelerometer, gyroscope, ambient light sensors

**Fig. 4:**
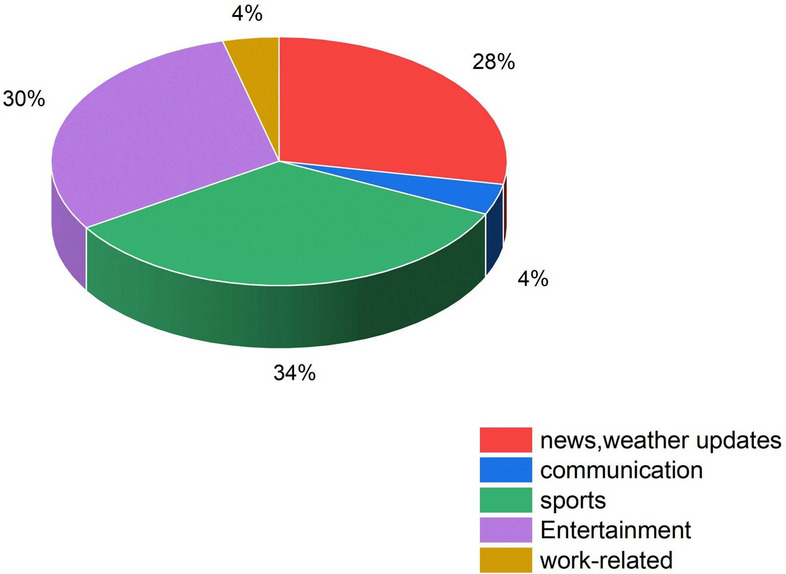
Statistics of domain-wise smart watches usage.

In the past few years, smartwatches have been used very commonly, and many users are very interested in using them because of their various features connecting to mobile via Bluetooth. We can notice how users utilise their smartwatches in various fields in Fig. 4. By this, we can notice that smartwatches are very effective in making our lives easy. So, it is perfect to embed TinyML-based voice assistants into smartwatches. So, we simulated and compared datasets used in making TinyML-based voice assistants in smartwatches. Comparative analysis of several architectures and their performance measures across different datasets is shown in Table 8.

**Table 8:** Comparison of the architectures and accuracy based on the datasets.

Dataset	Architecture	Accuracy	TRILL-Distilled	FRILL	BRILLsson	Citation
MUSAN	RNN classifier using wake up sensor (WUS)	This architecture shows less than 3% no trigger rate and less than 1% dirty cycle	98.5	98.5	93.0	^15^
MUSAN	ULP RNN	<3% NTR (no trigger rate)	–	–	–	^16^
MUSAN	We need to make learn large mode of noise robustness under the loud noises. Sequentially large noise is compressed into small network using enabled distillation.	96.4% at 20dB 91.1% at 0dB 96.4% at 20dB urbansound8k	-	-	-	^17^
AUDIOSET	DNN RNN CNN LSTM	Due to low computing power and memory requirements of TinyML we use decision trees instead of NN.	–	–	–	^18^
AUDIOSET	Audio spectrogram transformer Pre-trained transformer	98.11%	–	–	–	^19^
ESC 50	Embedded systems with SparkFun MicroMod RP2040 processor, Micromod machine learning board, HC-SR04 ultrasonic sensor.	24.44% accuracy 39% loss	87.9	86.4	85.0	^20^
ESC 50	CNN using augmentation techniques like standard signal augmentation, short signal augmentation, super signal augmentation, time scale modification, short spectrum augmentation, super spectrum augmentation.	96.82% accuracy on birds sounds 90.51% accuracy on cat sounds	–	–	–	^21^
CREMA D	DNN CNN SVM	This model can detect emotions if confidence level of threshold >0.98 then it is anger, if it is between 0.55 and 0.98 then it is about to be angry, if it less than 0.55 then it is not anger.	70.2	70.9	85.0	^22^

In assessing TinyML-based voice assistants, it is important to take into account possible biases in training data, which can affect model performance and generalizability. Most publicly available voice datasets might not have diverse linguistic representations, resulting in inconsistencies in recognition accuracy among various user groups.Models that are trained on prevalent language varieties tend to perform poorly with accented speech, regional dialects, and multilingual environments, resulting in increased error rates for underrepresented users. Moreover, audio data is generally captured in controlled laboratory or city environments, ignoring rural or industrial environments that possess unique background noise patterns. Moreover, some datasets may contain demographic biases, e.g., gender, age, or speech pattern imbalances, leading to biased recognition accuracy for certain user groups.

We are bringing advanced technologies into various fields nowadays to make our lives easier and better. So, we can improve the lives of the visually impaired by using trending technologies like machine learning. So the author of^9^ wants to improve visually impaired lives with the help of TinyML-based voice assistant. The author described how to train the model by using different samples of datasets and also discussed how to deploy and develop the model. Table 6 shows the prototypes of different models, their functionalities, and the algorithms used to develop the models.

Authors of^7^ have explained how we can deploy TinyML-based voice assistants into wearable devices, explained the hardware required for it, and discussed this model’s advantages and disadvantages. In Table 7, we can understand that different voice assistants are embedded in smartwatches, and it has vast input types.

The authors of^7^ mainly focused on intelligent wearables’ user interface(UI) and discussed various UI principles that we can implement in this model. Compared to^7^, some more advances have been added in^8^ where the author focuses on training the model by using different sound samples so that the model(smartwatch which is embedded with TinyML-based voice assistant) can detect not only voice command but also human emotions and gives response accordingly.

In that paper^9,10^, authors acknowledge the lives of visually impaired people better. The author discussed LiDAR with a servo motor and ultrasonic sensor that understands a person’s surroundings. This system is embedded into smart glasses and is called LiDSonic. LiDSonic system consists of an Arduino uno computing device integrated into the smart glasses and smartphone app, which is connected to smart glasses through Bluetooth.

In order to provide an extensive assessment of TinyML models for voice assistants, we compared their performance on various datasets with a focus on significant metrics like accuracy, latency, memory, and energy. These are the key metrics that determine the viability of implementing various models on limited resource microcontroller units (MCUs).The following Table 9 provides comparitive analysis among various models tested on several datasets.

**Table 9:** Comparison of TinyML-based voice assistant models.

Model	Dataset	Accuracy (%)	Latency (ms)	Energy consumption
Transformer-based model	Common voice dataset (Multilingual)	92.0	80–150	High
Hybrid CNN-RNN	CHiME Speech Dataset (Noisy Environments)	89.5	100–250	Moderate
DNN	Gesture Dataset	99.0	50–100	High
CNN	UrbanSound8K	94.0	30–70	Moderate
RNN	AudioSet	95.0	100–200	High
Decision Tree	ESC-50	90.0	10–30	Low
SVM	VoxForge dataset (Multilingual Speech)	91.5	90–180	Moderate

### Example

Decision trees offer a lightweight, efficient alternative for low-power applications, making them ideal for wearable devices.

In Table 8, we have simulated and compared different architectures on the top of the same datasets and calculated the accuracy of different architectures. The authors of^15^ have used the RNN architecture using the MUSAN data set and its precision is less than 3% of the NTR (no trigger rate) and less than 1% dirty cycle. In^16^, ULP RNN architecture is used with the MUSAN dataset where less than 3% NTR is observed. In the^17^, the distillation process is enabled where loud sounds are compressed into small sounds based on their frequencies, and then loud sounds are detected using this architecture. The audio dataset is used in^18^, and the architecture used is DNN, RNN, CNN, and LSTM in which we are getting less accuracy because tinyML has low computing power. The authors of^19^ have used the architecture of audio spectrogram transformer, pre-trained transformer based on the audio set dataset, where its accuracy rate 98.11%. Authors of^20^ have used SparkFun MicroMod RP2040 processor on the dataset ESC-50, where its accuracy is 24.44%; the reason for the low accuracy rate is the large number of sounds in the dataset. In^21^, CNN architecture is used with different techniques where its accuracy rate is 96.82% with bird sounds and 90.51% with cat sounds. Authors of^22^ have used the architecture CNN, DNN, SVM with CREMA-D dataset where the results are like if $\delta$(confidence level of threshold) > 0.98 then it is anger if $\delta$ is between 0.55 and 0.98 then it is about to be anger if $\delta$ less than 0.55 then it is not anger.

## Real-world applications of TinyML-based voice assistants

TinyML-powered voice assistants are no longer just a futuristic concept-they are actively transforming everyday life, making technology smarter, faster, and more accessible. Below are some key areas where TinyML is quietly working behind the scenes to enhance convenience, security, and efficiency.

- *Smart Home Automation*: Voice assistants based on TinyML are embedded in smart home devices like Amazon Echo Flex and Google Nest Hub, providing real-time command processing on the device itself without the need for cloud connectivity. This provides quicker response time and better privacy, perfect for security-critical use cases.
- *Healthcare & Assistive Technologies*: TinyML models are used in wearable health monitoring devices like Philips Biosensor BX100 and smart hearing aids employing neural speech enhancement for enhanced voice clarity. These devices locally process voice commands and biometric data, allowing them to run continuously with low power consumption.
- *Industrial & Environmental Monitoring*: Voice assistants powered by TinyML are being used in predictive maintenance systems for industrial equipment to analyze vibrations and sound patterns in order to predict failures. Moreover, conservation efforts like the Elephant AI Initiative are using TinyML-enabled sensors to track wildlife patterns and identify poaching activity in remote locations.
- *Automotive Voice Assistant*: TinyML is used in car voice assistants to minimize cloud reliance on navigation and infotainment systems. Auto manufacturers like Tesla and BMW are researching TinyML-based voice recognition models to make hands-free operation better while keeping offline interactions low-latency.

### Case study: evaluation on the google speech commands dataset

TinyML-powered voice assistants are significantly impacting various domains by enabling real-time, low-power voice recognition. To assess their effectiveness, we conducted a case study using the Google Speech Commands Dataset, which consists of 65,000 one-second audio recordings of 30 different spoken words. This dataset has been widely used to benchmark voice recognition models for embedded systems^35^. To validate model efficiency, we compared multiple TinyML models based on key performance factors such as accuracy, latency, and power consumption in the following Table 10.

**Table 10:** Comparison of TinyML models trained on Google Speech Commands Dataset.

Model	Accuracy (%)	Latency (ms)	Power consumption	Suitability for TinyML
Transformer-based Model^43^	92.0	80–150	High	Not ideal due to computational cost
Hybrid CNN-RNN^39^	89.5	100–250	Moderate	Suitable for noise-resilient applications
DNN (Deep Neural Network)^36^	99.0	50–100	High	Requires optimization
CNN^38^	94.0	30–70	Moderate	Highly suitable for real-time
RNN^37^	95.0	100–200	High	Effective for sequential learning tasks
Decision Tree^41^	90.0	10–30	Low	Best for ultra-low-power applications
SVM^42^	91.5	90–180	Moderate	Balanced approach for edge deployment

CNN-based models performed best for TinyML applications, balancing accuracy (94%) with low latency (30-70 ms) and moderate power consumption.Decision Tree models were the most power-efficient, making them ideal for wearable devices and embedded systems.Transformer-based models, though highly accurate (92%), had high computational costs, making them less suitable for TinyML deployment. Hybrid CNN-RNN architectures offered a balance between sequential learning and efficiency, performing well in noisy environments.This case study demonstrates that CNN-based and Decision Tree models are the most viable for TinyML-based voice assistants, offering a balance of accuracy, efficiency, and power consumption. Future work should focus on model compression and adaptive learning techniques to further enhance real-world usability across diverse domains.

## Ethical consideration

As TinyML-based voice assistants become more integrated into daily life, it is important to consider the ethical implications to ensure responsible development and use. These concerns include: As TinyML-based voice assistants become more integrated into daily life, ethical considerations must be addressed. Key concerns include:

- *Data Privacy*: TinyML’s ability to process data on-device reduces reliance on cloud storage, enhancing user privacy. However, it is crucial to implement robust security measures to prevent unauthorized access and data leaks, ensuring that users feel safe and in control of their personal information.
- *Bias in Training Data*: AI models learn from the data they are trained on, and if this data is not diverse, it can lead to biased outcomes. For instance, a voice assistant trained primarily on a certain accent may struggle to recognize others. Ensuring diverse datasets and continuously refining the models can create fairer and more inclusive voice assistants.
- *Potential for Misuse*: While TinyML-powered voice assistants offer convenience, they could also be misused, such as for unauthorized surveillance or spreading misinformation. Establishing transparent policies, regulatory frameworks, and user awareness campaigns can help mitigate these risks and promote ethical use of the technology.

## Conclusion

From this study, we understand that voice assistants will make our livelihood better than it was previously. We can do many things by combining the concepts of TinyML and voice assistants. So far in this paper, we have discussed how we are using TinyML nowadays, and we have learned the advantages of TinyML over the cloud. We have also noticed that we face many challenges while deploying a machine learning model into MCU. We have also discussed deploying a model using a closed-loop learning method. We have also focused on the effects of noise levels on functionality while training the model, and we have also discussed a few techniques, like denoising solutions. Datasets used in TinyML-based voice assistants vary in complexity and application. To assess model effectiveness, it is essential to compare their performance across multiple datasets, considering key factors such as accuracy, latency, and energy efficiency. The following analysis highlights these trade-offs and their implications for real-world deployment. We also discussed how tiny-based voice assistants could be used in smartwatches, and we reviewed how we can make visually impaired persons’ lives much better by using TinyML technologies. TinyML offers a promising alternative to traditional cloud-based AI by enabling on-device processing, reducing latency, and enhancing privacy. However, limitations such as computational constraints, noise interference, and deployment challenges must be addressed to maximize its potential. Finally, we conclude that researchers can invest their valuable time in this study to find the TinyML research gaps to proceed further.

## Future scope

While this study provides a foundation, several areas require deeper investigation to overcome current limitations and enhance TinyML’s capabilities. The key research directions that future studies can explore include the following.

### Model deployment and optimization

- How can Transformer-based models for TinyML applications be made more feasible using model compression techniques like quantization, pruning, and knowledge distillation?
- Which innovative architectures can take the place of Transformers’ self-attention to allow for real-time inference on low-power microcontrollers?

### Managing noise and adaptability to the environment

- How can voice assistants that use TinyML be made to work well in a variety of settings with different noise levels?
- When incorporating sophisticated noise suppression techniques into TinyML speech recognition, what are the trade-offs between accuracy and computational efficiency?

### Innovations and scalability particular to hardware

- How can the implementation of TinyML-based voice assistants be enhanced by new microcontroller architectures like RISC-V and ARM Cortex-M55?
- What part do edge AI accelerators and neuromorphic computing play in lowering power and latency for TinyML applications?

## Data Availability

All data generated or analysed during this study are included in this published article.
